# Anesthesia by Rapid Breathing

**Published:** 1899-03

**Authors:** W. G. A. Bonwill

**Affiliations:** Philadelphia, Pa.


ARTICLE IV.

### ANESTHESIA BY RAPID BREATHING.

DR. W. G. A. BONWILL, PHILADELPHIA, PA.

It is diversion of the will power, for when the lungs are being inflated violently, the will cannot take recognizance of actual pain. It is for the instant complete. Let any of you hurt a finger and how soon it is put in the mouth and a violent inhalation taken several times until pain is relieved. The infant is crying violently, while in pain from an accident, is relieved and falls to sleep from the constant sobbing and increased respiration. All temporary teeth I extract by this one sudden inhalation or diversion of the will, without a tear nor complaint. Two or three can be extracted while the breath is held in the lungs.

This led to the discovery that rapid breathing for sixty to ninety seconds, at the rate of 100 to the minute, would produce such an obtunity of the nerves and nerve centers that analgesia would be produced and the patient still be conscious of touch. This enabled me to perform all excavating—extracting both teeth at the bony pulp, as well as all minor operations in surgery. A single respiration quickly taken and the breath held in the lungs will suffice for many trifling operations and all that is needed in extracting the temporary and permanent teeth of children. This was the birth of analgesia, in 1856, as proven upon my own person while operating upon myself.

This property of all anesthetics is very little known by medical men, and they have missed a very important part of anesthesia. Analgesia should be better understood and the application of rapid respiration would come into use in thousands of minor operations where the custom is to completely anesthetize the subject. Let surgeons and dentists learn how to produce this effect, and ether, chloroform and gas would no longer be used in three fourths of the cases. In midwifery it is all that is desirable.

Let any one imagine that they are blowing a fire to make it blaze, taking full inhalations and forcibly ejecting or blowing, which can be done at least 100 times a minute, and while the patient is doing this the operator should talk to him, say no pain will be produced, and urging him to breathe in this manner until sixty seconds have passed, when the patient can not feel any pain, as the voluntary system is subdued. The pneumogastric nerve now says, you can not go on as life would become extinct. For the next three minutes the respiration is only about six to the minute, showing the blood has been over-oxygenated. The patient has to be urged to breathe. When this is done previous to using ether or chloroform only one-half the quantity is used, the effect is more profound, and there is not as much danger. Since this discovery of the rapid breathing method, in 1872, although analgesia was discovered by me in 1856, and was the stepping-stone thereto, I have used no other means of painless surgery in dentistry.

Cataphoresis is not a fact as applied to sensitive dentine, and should have no place in dentistry.—*Dental Brief.*

